# Effects of Timing of Grazing on Arthropod Communities in Semi-Natural Grasslands

**DOI:** 10.1673/031.010.6001

**Published:** 2010-06-10

**Authors:** Lisette Lenoir, Tommy Lennartsson

**Affiliations:** ^1^ Department of Ecology and Environmental Research, Swedish University of Agricultural Sciences, Box 7072, Uppsala, S-750 07 Sweden; ^2^ Swedish Biodiversity Centre, Swedish University of Agricultural Sciences, Box 7007, Uppsala, S-750 07 Sweden

**Keywords:** Aranidae, Carabidae, Formicidae, semi-natural pasture, timing of grazing

## Abstract

Arthropod communities were investigated in two Swedish semi-natural grasslands, each subject to two types of grazing regime: conventional grazing from May to September (continuous grazing) and traditional late management from mid-July (late grazing). Pitfall traps were used to investigate abundance of carabids, spiders, and ants over the grazing season. Ant abundance was also measured by mapping nest density during three successive years. Small spiders, carabids and ants (*Myrmica* spp.) were more abundant in continuous grazing than in late grazing while larger spiders, carabids, and ants (*Formica* spp.) were more abundant in late grazing. The overall abundance of carabids was higher in continuous grazing in the early summer but higher in late grazing in the late summer. The switch of preference from continuous to late grazing coincided with the time for larvae hibernating species replacing adult hibernating. We discuss possible explanations for the observed responses in terms of effects of grazing season on a number of habitat variables for example temperature, food resources, structure of vegetation, litter layer, competition, and disturbance.

## Introduction

Seminatural grassland is one of the most species rich habitats in Europe's open landscapes. Long continuity of grazing or mowing, without application of fertilisers and pesticides (Pärt and Söderström 1998) has built up a high diversity of plants and insects (Appelqvist et al. 2001). For example, Swedish grasslands can harbour up to 60 species of vascular plants per m^2^ (Eriksson and Eriksson 1997).

During the 20^th^ century, agriculture became more intensive in Europe and traditional land use practices such as grazing, mowing, and burning were abandoned. Many areas of grassland have thus become overgrown, while for many organisms the farmland that remains has been rendered unsuitable by the use of fertilisers and pesticides (Anthelme et al. 2001; Watkinson and Ormerod 2001; Firbank 2005; Hole et al. 2005; Schmidt et al. 2005). Most countries in Western Europe have lost more than 95% of their original grassland areas (e.g. Statistiska Centralbyrån 1990; Nature Conservancy Council 1984; Kumm 2003). Abandonment of grazed fields has been identified as an important cause of the decline of grassland biodiversity (Karlsson 1984; Fuller 1987). As a consequence, large numbers of red-listed species are associated with these habitats (Gärdenfors 2000).

Most temperate grasslands are dependent on regular disturbances that counteract the succession towards scrubland and eventually forest. The nature of this disturbance, for example in terms of type, timing, and intensity, is essential for the grasslands' biodiversity. In most European countries, the grasslands have a long management history with grazing and mowing as the dominating disturbance regimes (Poschlod and Bonn 1998; Söderström et al. 2001; Eriksson et al. 2002). Therefore, grassland biodiversity should be favoured by management that is as similar as possible to the local historical management regimes (Lennartsson and Ostermeijer 2001). However, the present management methods often differ considerably from traditional management (Gustavsson 2007; Dahlström et al. 2006, 2008). One important change in management is the decreased use of late-season management (García 1992; Beaufoy et al. 1995; Ihse and Lindahl 2000). Earlier, about 20–30% of the semi-natural grassland area was subject to late season (in Sweden from mid-July at the earliest) management. Now, only approximately 3% is late grazed (Dahlström et al. 2008).

Management experiments have shown that type and timing of management have profound effects on grassland biodiversity (Morris 2000) and have also indicated that the present management may not provide sufficient conditions for grassland biodiversity (Zobel 1992; Poschlod et al. 2005). The main ecological effect of late management by mowing or late grazing is that the vegetation is left undisturbed in the early summer. This is advantageous for seed production, especially in plants with early reproduction (Karlsson 1984; Zopfi 1993; Lennartsson and Svensson 1996; Simán and Lennartsson 1998). Timing of grazing affects the vegetation structure and has been shown to also affect the species composition and abundance of ants (Boulton et al. 2005), beetles (McFerran et al. 1994) and spiders (Dennis et al 2001; Schwab et al. 2002).

Studies of ecological effects of grazing and other grassland management often use one or a few species, usually vascular plants, as indicators for the grassland condition (Lennartsson and Oostermeijer 2001), but few studies analyse effects on different taxa or taxonomic groups. Some studies have indicated that different species groups in semi-natural grasslands may differ considerably regarding which management regime is optimal (e.g. Söderstöm et al. 2001; Vessby et al. 2002).

In this study, conventional grassland management, i.e. grazing from May to September, was compared with an experimentally applied traditional management, grazing from mid-July. The effects of grazing regime were analyzed regarding abundance, species richness, and species composition of different groups of predator arthropods: ants, Carabid beetles and spiders.

## Materials and Methods

### Study sites

The study was conducted in two seminatural pastures in south-central Sweden: Pustnäs, 2 hectares, 59° 45′ N, 17° 45′ E; and Harpsund, 12 hectares, 59° 05′ N, 16° 29′ E. In both pastures, the mean annual precipitation was about 600–700 mm, and the mean annual temperature was about 7° C. The pasture at Pustnäs is located in a flat area, whereas the Harpsund pasture consists of a low east-west stretched ridge. The vegetation type at both sites was mainly dry to mesic herb-rich *Agrostis capillaris* L. (Poales: Poaceae) meadow (Påhlsson 1994). Other dominating species were *Poa pratensis* L. (Poaceae), *Filipendula vulgaris* Sturm (Rosales: Rosaceae), *Leontodon autumnalis* L. (Asterales: Asteraceae), *Leucanthemum vulgaris* Lam. (Asteraceae), *Lotus corniculatus* L. (Fabales: Fabaceae), *Prunella vulgaris* L. (Lamiales: Lamiaceae), *Ranunculus* spp., and *Trifolium* spp. Apart from the experimental areas (see below) both sites were grazed annually from May to September by about 1.8 (Pustnäs) and 1.2 (Harpsund) steers or heifers per hectare.

### Experimental design

Two homogenous (by means of vegetation) areas in each pasture were chosen, and an alternative grazing regime was established by separating, by fencing, one area of 1 hectare (Pustnäs) and one of 4 hectares (Harpsund) from the continuously grazed pastures. Data sampling was performed in these exclosures and in the continuously grazed grassland adjacent to the exclosures. The exclosures were not grazed until 27 July in Harpsund and 18 July in Pustnäs, when the fence was opened and the grazers were allowed to utilize the whole pasture. The alternative grazing regimes were initiated in 1997 in Pustnäs and in 2001 in Harpsund and were applied each year until 2005. The difference in time of opening was due to practical reasons related to the farmers' cattle management and arrangement of grazing.

### Vegetation height and litter depth

Vegetation height was measured using a rising plate (Sanderson et al. 2001) at 30 random sampling points per grazing treatment at 6–9 occasions from late May to late September 2001–2003. Litter layer thickness, from the litter surface to the mineral soil, was measured at 30 random points on the first sampling occasion, using a mm-graded stick.

### Temperature data

Temperature data on the mean of each 24 hour-period throughout the study period were provided by the Ultuna Climate and Bioclimate station (see http://www.grodden.evp.slu.se/slu_klimat/station.html).

### Species composition and abundance of ants, carabid beetles, and spiders

Arthropods were sampled by using pitfall traps: 850 ml plastic jars, 12 cm in diameter buried to the level of the ground surface. The traps were filled 1/3 with water and a drop of detergent to reduce the surface tension.

In Harpsund, 28 traps were installed in each grazing treatment in a spatial arrangement that covered the environmental variation within each treatment area. In each grazing treatment, 7 traps were located uphill and 7 downhill on the north-facing slope of the ridge, and 7 traps were located uphill and 7 downhill on the south-facing slope. Hereafter, a group of 7 traps is called a block. The distance between the traps was at least 10 m, and the distance between the blocks was at least 20 m.

The grassland in Pustnäs was smaller than in Harpsund, and, therefore, only 7 traps per grazing treatment were randomly established.

The traps operated for 10 periods during 7 days from 13 May to 28 August 2002 in Harpsund and nine periods from 30 May to 29 August 2002 in Pustnäs. The traps operated during 7 successive days, followed by 7 days of non-operation. Animals were collected from each trap after each operation sequence and preserved in 50% propylenglycol. All beetle samples from Pustnäs before 10 July were accidentally destroyed in the lab, leaving seven sampling periods for carabids from that site.

Ants were identified to species level based on Seifert (1996), and beetles to species level based on Lindroth (1985, 1986). Due to resource limitation, spiders were collected only at five (Harpsund) and four (Pustnäs) sampling events. Spiders were identified to species, genus, or family level using Roberts (1995) and Jones-Walters (1994).

### Ant mounds

To investigate the effect of grazing regime on density and persistence of ant nests, and to investigate the occurrence of *Lasius flavus* Foerster (Hymenopera: Formicidae), which is not easily caught in pitfall traps, all hillocks taller than 10 cm were mapped. In Pustnäs, mapping was performed over the whole 1 hectare treatment areas. In Harpsund, nests were mapped in one 0.04 ha area per treatment, placed 10 m from each other, at opposite sides of the fence. Ants inhabiting the mounds were collected and determined to species level, and mounds without ants were assigned abandoned. Mapping was done in July in 2002, 2003, and 2004 in Pustnäs and in 2002 and 2003 in Harpsund. Height and diameter of the mounds was measured in 2002.

### Statistical analyses

The sampling design in Pustnäs and Harpsund was not identical, and the two sites were therefore analyzed separately.

### Pitfall traps

For the data from Harpsund, variation in capture efficiency attributable to the individual locations of the traps was avoided by pooling the catches from the 7 traps in each block at each trapping occasion. Thus, the estimate of species richness was based on the number of species found in each block at one occasion in one treatment. In Pustnäs, no blocks were used and species richness was based on individual traps. The species of Carabid beetles and spiders were also analyzed in terms of functional groups, and since body size can be assumed to be important for several aspects of a species' ecology (see discussion), the grouping was partly based on size. Based on the size frequencies beetles were classified in three size classes, < 5 mm, 5–8 mm, and > 8 mm and according to life-cycle, habitat preference, and food preference (Table 1) (Lindroth 1992). Life-cycle refers to which stage that hibernates and was used since this can be assumed to influence the phenology of the species, in turn potentially important for the species response to grazing season. Spiders were classified in six classes according to a combination of size and foraging behavior (web-builders < 3 mm, web-builders 3–6 mm, runners < 6 mm, runners 6–10 mm, runners > 10 mm, and “sit-and-wait-species”, Roberts 1995), and in three taxonomic groups of wolf spiders (Lycosidae), *Paradosa* spp, *Alopecosa* spp, and *Trochosa* spp. In order to meet assumptions of normality, all data on spiders and beetles were log (n + 0.1) transformed before analysis.

Ants are social insects, while spiders and beetles are not and this affectd the numbers of individuals trapped. Worker ants often follow one another so the actual number of individuals trapped in pitfalls is not related to the density of ant colonies. Therefore, colony density of a species in a block at one sampling occasion in Harpsund was estimated as the proportion of traps in the block that contained the species. Due to the chosen distance between traps, this proportion provides a good approximation of the colony density of small species with limited movement ranges (e.g. *Myrmica* spp., *Lasius* spp.) and of the activity density of bigger species (e.g. *Formica* spp.) with larger movement ranges (Savolainen et al. 1989). Activity density of smaller species was estimated for both Harpsund and Pustnäs as the number of individuals trapped (per trap in Pustnäs, per block in Harpsund). In order to meet assumptions of normality, data on colony density were arcsine transformed (Fowler et al. 1998) and those on activity were log (n + 0.1) transformed.

**Table 1.**
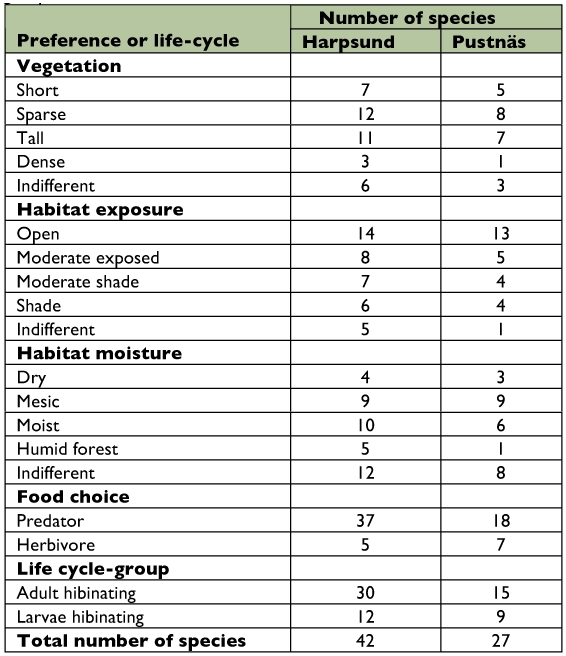
Total number of species found in two seminatural grasslands, Harpsund and Pustnäs, divided in different preference or life-cycle groups.

It was assumed that the difference in population size of the arthropods between the two treatment areas varied over time and that the similarities at different times before onset of late grazing were larger than the similarities between before and after the onset of late grazing. Therefore, the data sets of Harpsund and Pustnäs were divided into (1) those that includes all observations before onset of late grazing, (2) those including all observations after onset of late grazing, and (3) those including observations on 26 July, one sampling day before, and 12 August, the sampling day after, the onset of late grazing. The Shapiro-Wilk's test for normality was used to test the appropriateness of the statistical model. For Harpund, repeated measures data on arthropods were analysed using a mixed effects model with grazing regime as fixed factor, block as random factor, and sampling time as repeated factor (Littell et al. 2006). For Pustnäs, repeated measures data on arthropods were analysed using a mixed effects model with grazing regime as fixed factor, trap as random factor, and sampling time as repeated factor.

**Figure 1.**
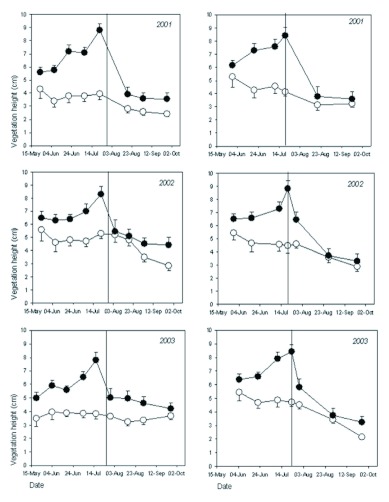
Vegetation height over three grazing seasons in two grazing regimes, continuous grazing (open circles) and late grazing (filled), in two seminatural pastures, Harpsund (left panels) and Pustnäs (right). The late onset of grazing is indicated by a vertical line. Error bars show one S.E., and for clarity (to avoid overlap), one-sided error bars are used. High quality figures are available online.

Bonferroni or similar corrections for multiple tests were not applied, but instead the results were interpreted with care, focusing on single results instead of the number of significant differences (e.g. Lindberg and Bengtsson 2006).

**Figure 2.**
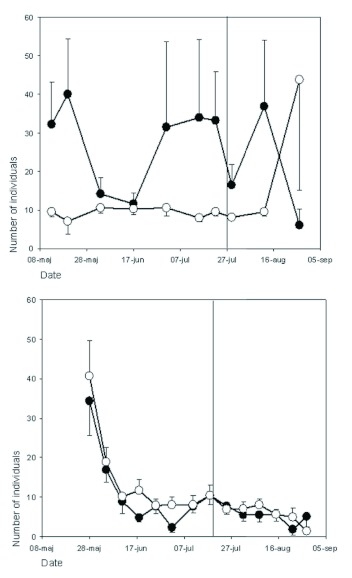
Mean numbers of individuals of ants (Hymenoptera) caught by pitfall trapping over the growing season in two semi-natural grasslands, Harpsund (top panel) and Pustnäs (bottom), subject to two grazing regimes, continuous grazing (open circles) and late onset of grazing (filled). The late onset of grazing is indicated by a vertical line. Error bars show one S.E., and for clarity (to avoid overlap), one-sided error bars are used. High quality figures are available online.

### Ant mounds

The total, not mean numbers of ant mounds per treatment, was counted. Therefore, contingency tests, G-tests, were used to test for differences between grazing treatments in number of inhabited and abandoned ant mounds, respectively. Each year and site was analyzed separately. The height of inhabited ant mounds was analyzed for each site separately by two-way ANOVA with ant species (*Lasius niger* and *L. flavus* (Formicidae)) and grazing regime as factors.

### Vegetation data

Due to non-normality, tests on vegetation height and litter depth were performed using non-parametric tests.

## Results

### Vegetation height and litter depth

In both pastures the vegetation in continuous grazing was reduced to 3–5 cm in early June, and this height was kept rather constant until late July, when vegetation height was further reduced, to a height of 2–3 cm in late September (Figure 1). In late grazing, vegetation grew to a height of 8–9 cm until onset of late grazing. After onset of late grazing, vegetation was rapidly reduced but remained 0.5–2 cm taller than in continuous grazing throughout the season (Figure 1). By the end of the season, vegetation height in Harpsund differed about 2 cm between the grazing regimes; in Pustnäs vegetation height differed less than 1cm.

Thickness of litter layer in early June in Harpsund was 4.6–6.8 mm in continuous grazing from 2001 to 2003 and 8.7–11.0 in late grazing from 2002 to 2003. This difference between treatments was significant in both years (Mann-Whitney U-test, p < 0.05). In 2001, i.e. at the beginning of the first experimental season, litter thickness did not differ between treatments in Harpsund (6.8 ± 3.0 and 6.2 ± 3.2 in continuous and late grazing, respectively). In Pustnäs, litter layer varied between 4.2 and 5.5 mm during 2001–2003 in continuous grazing and between 8.8 ± 2.8 and 11.3 ± 3.4 in late grazing, and the difference between treatments was significant for all three years (p < 0.05).

### Ants

Number of individuals: In Harpsund, 8750 individuals belonging to 15 different species were trapped, and, in Pustnäs, 2204 individuals of 11 species. Pitfall traps detected a significant overall effect of grazing regime on the number of individuals in Pustnäs but not in Harpsund. Before onset of late grazing, more individuals were found in continuous grazing in Pustnäs (repeated measures ANOVA; F_1,112_ = 6.94, p = 0.02), but the magnitude of difference varied over time (Figure 2). In Harpsund, there was a tendency for the opposite pattern, but because of high variation, no significant effects could be detected (Figure 2). In Pustnäs, the ants were most active in the end of May; in Harpsund, they were most active in mid-June (Figure 2). *L. niger* was the most numerous ant species in both Pustnäs and Harpsund, but none of the measurement methods detected significant effects of grazing regime on the activity of this species.

Number of species and species-specific responses: In Harpsund, pitfall traps showed that species richness was not affected by grazing regime. The ant, *Myrmica rubra* L. was present in larger numbers (repeated measures ANOVA; F_1,64_ = 8.7, p = 0.03) and had higher colony density (F_1,64_ = 9.8, p = 0.02) in continuous grazing, whereas *Formica polyctena* had higher activity density in late grazing (F_1,64_ = 12, p = 0.04). The colony density, but not the abundance of individual *Myrmica scabrinodis* Nylander ants, was higher in late grazing before (F_1,64_ = 12.5, p = 0.04), but not after, late onset of grazing. The total number of individuals of *Myrmica* spp. was higher in continuous grazing (F_1,32_ = 22, p = 0.009).

In Pustnäs, pitfall traps showed higher species numbers in continuous grazing compared to late grazing (repeated measures ANOVA; F_1,112_ = 8.14, p = 0.008). The total number of individuals of *Myrmica* spp. was significantly higher in continuous grazing (F_1,112_ = 9.8, p = 0.005), and this effect was also found for two of the *Myrmica* species, *M. lobicornis* and *M. rubra* (p < 0.04 both species). In contrast, *Formica rufibarbis* F. was mostly present in late grazing (F_1,112_ = 22, p < 0001). After onset of late grazing, these differences between grazing regimes persisted.

**Figure 3.**
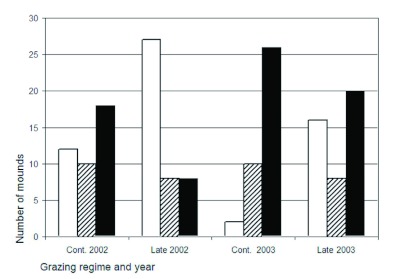
Number of ant mounds during two years and in two grazing regimes, continuous grazing (Cont.) and late onset of grazing (Late). White bars show *Lasius flavus* mounds; dashed bars show *Lasius niger* mounds, and black bars show abandoned mounds, sometimes invaded by *Myrmica* spp. High quality figures are available online.

Ant mounds: In late grazing in Harpsund, 27 mounds of *L. flavus* and 8 of *L. niger* were mapped, corresponding to 675 and 200 per hectare, respectively. In continuous grazing, 2 mounds of *L. flavus* and 10 of *L. niger* were mapped, corresponding to 300 and 250 mounds per hectare, respectively. Thus, more ant mounds than expected by random allocation were inhabited by *L. flavus* in late grazing than in continuous grazing (G-test; p < 0.05 in both years, Figure 3), while for *L. niger* a non-significant opposite pattern was found. In Pustnäs, only one mound of *L. flavus* per treatment area was found, and 25 and 29 mounds of *L. niger* were found in late and continuous grazing, respectively. In Harpsund, 98% of the inhabited mounds mapped in 2002 were still inhabited in 2003. The number of mounds inhabited by *L. flavus* decreased between 2002 and 2003 in both pastures, while the number of abandoned mounds or mounds inhabited by *Myrmica* spp. increased over time (Figure 3). Grazing regime had no significant effect on the mean height of the anthills (17.4 ± 5.4 and 14.4 ± 3.8 cm in late and continuous grazing, respectively; two-way ANOVA, F_1,23_ = 0.9, p = 0.4).

In Pustnäs, more than half of the mounds that were found in 2002 were completely destroyed by cattle in 2003 and 2004. No significant effects of grazing regime were found on the number of mounds inhabited by *L. niger*, nor on the number of abandoned mounds was found. Grazing regime had no significant effect on the mean height of the anthills (16.9 ± 5.4 and 17.9 ± 5.8 cm in late and continuous grazing, respectively).

**Figure 4.**
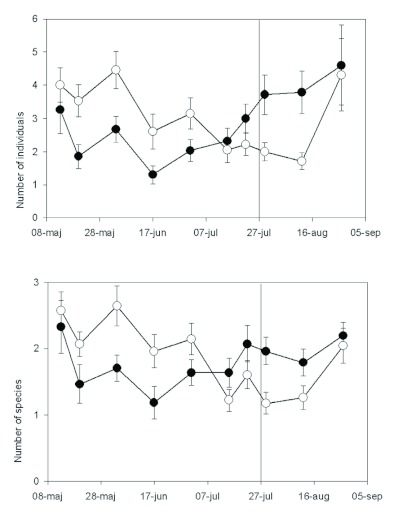
Mean numbers of individuals and species of ground beetles (Coleoptera: Carabidae) caught by pitfall trapping over the growing season in a semi-natural grassland in Harpsund that was subject to two grazing regimes: continuous grazing (open circles) and late onset of grazing (filled). The late onset of grazing is indicated by a vertical line. Error bars show one S.E., and for clarity (to avoid overlap), one-sided error bars are used. High quality figures are available online.

### Spiders

In Harpsund, 7502 specimens belonging to eight families were counted. Of the Lycosidae, 13 different species were identified. Grazing regime had no significant effect on number of individuals. Analysis of functional groups showed that the number of individuals of web builders < 3 mm was higher in continuous grazing after onset of late grazing (repeated measures ANOVA; F_1,16_ = 7, p = 0.04), but not earlier in the summer. Runners > 10mm, mainly *Trochosa terricola* Thorell (Araneae: Lycosoidea), were more common in late grazing after 27 July (F_1,16_ = 6.1, p = 0.048), but not earlier in the summer. Grazing regime had no significant effects on the number of individuals or species of the genera *Paradosa* spp., *Alopectosa* spp., or *Trochosa* spp. Some single species showed differences between grazing regimes at some sampling dates in Harpsund, but these differences varied in an inconsistent manner.

In Pustnäs, 2465 specimen belonging to seven families were counted. Of the Lycosidae, 11 different species were identified. More species were found in late compared to continuous grazing before onset of late grazing, i.e. in the undisturbed vegetation (F_1,42_ = 22, p = 0.0002). After onset of late grazing, no differences were found. Analysis of functional groups showed that the number of individuals of web builders < 3mm was higher in continuous grazing (F_1,42_ = 60, p < 0.0001) both before and after onset of late grazing. In contrast, the abundance of sit-and-wait-species and runners > 10mm (*Trochosa* spp.), was about seven times higher in late grazing (F_1,42_ = 60, p = 0.0006) both before and after onset of late grazing. The taxonomic group *Linyphiidae* was about four times higher in continuous than in late grazing (F_1,42_ = 60, p = 0.0003). No other taxonomic groups were significantly affected by grazing regime. Of single species, the small runner, *Paradosa fulvipes* was caught in higher numbers in late grazing (F_1,42_ = 60, p = 0.007).

### Carabid beetles

Number of individuals and species: In Pustnäs, a total of 288 individuals belonging to 27 species and, in Harpsund, 1429 individuals of 42 species were trapped during the study.

Grazing regime as main factor had no significant effect on either species richness or number of individuals (repeated measures ANOVAs; Harpsund, p > 0.1; Pustnäs, p > 0.4). Analyses of cumulative data showed, in contrast, that the abundance of individuals was about 1.5 times higher in late grazing in Pustnäs (F_1,14_ = 5.1, p = 0.04). This effect was not found in Harpsund.

**Figure 5.**
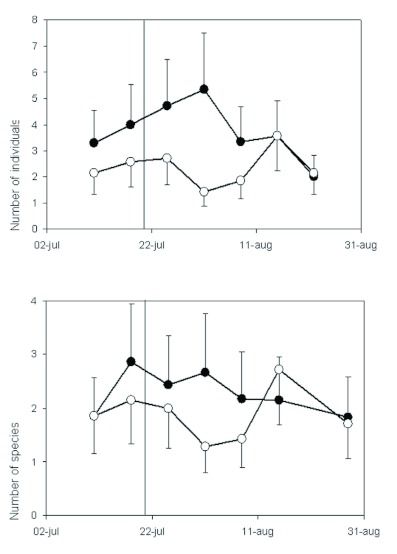
Mean numbers of individuals and species of ground beetles (Coleoptera: Carabidae) caught by pitfall trapping over the growing season in a semi-natural grassland in Pustnäs that was subject to two grazing regimes: continuous grazing (open circles) and late onset of grazing (filled). The late onset of grazing is indicated by a vertical line. Error bars show one S.E., and for clarity (to avoid overlap), one-sided error bars are used. High quality figures are available online.

In Harpsund, however, the number of *Carabidae* individuals before onset of late grazing was significantly affected by the grazing regime/date interaction (repeated measures ANOVA; F_64,7_ = 2.39, p = 0.04). For a number of species, this interaction was marginally significant (F_64.7_ = 2.04, p = 0.08). In general, the number of species and individuals were higher in continuous grazing in the early summer and higher in late grazing in the late summer (Figure 4). The shift in preference between the two treatment areas occurred around 1 July, before the onset of late grazing (29 July). In Pustnäs, no interaction effect was found for either number of species (p = 0.6), or number of individuals (p = 0.5, Figure 5).

**Figure 6.**
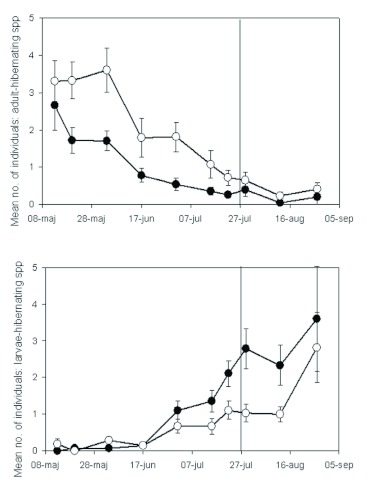
Mean numbers of individuals of adult-hibernating (upper panel) and larvae-hibernating (lower panel) species of ground beetles (Coleoptera: Carabidae) caught by pitfall trapping over the growing season in a semi-natural grassland in Harpsund that was subject to two grazing regimes: continuous grazing (open circles) and late onset of grazing (filled). The late onset of grazing is indicated by a vertical line. Error bars show one S.E., and for clarity (to avoid overlap), one-sided error bars are used. High quality figures are available online.

Responses of species with different life cycles: In Harpsund, species with different life cycles showed somewhat different grazing regime preference (Figure 6). In downhill blocks, the number of individuals of adult-hibernating species tended to be higher in continuous grazing before, but not after, late onset of grazing (repeated measures ANOVA; F_32,1_ = 13, p = 0.06). In the uphill blocks, a significant grazing regime/date interaction was found for adult-hibernating species (F_32,7_ = 4.2, p = 0.04), and the number of individuals was higher in continuous grazing at most dates. The adult-hibernating species were replaced by larvae-hibernating species during the first half of July (Figure 6), and date had, consequently, a significant effect on the number of individuals of both life cycle types (p < 0.0001). Of the species that were found in sufficient numbers to be analyzed separately, the adult hibernating species, e.g. *Amara communis* Panzer (Coleoptera: Carabidae), *Bembidion guttula* F., *Bembidion Lampros* Herbst, and *Pterostichus versicolor* Sturm, were more common in continuous grazing early in the summer in Harpsund. Around 1 July, those species disappeared and were replaced by larvae hibernating species, such as *Calathus fuscipes* Goeze, *Harpalus latus* L., *Pterostichus niger* Schaller, and *Trechus secalis* Paykull, which were more common in late grazing. With few exceptions, no significant grazing regime preference could be detected at the species level.

Adult-hibernating species were considerably smaller (6.7 ± 3.1 mm) than larvae-hibernating species (10.1 ± 5.5 mm). Body size of imagines explained, however, little of the variation in individual number between grazing regimes in Harpsund. The grazing regime/date interaction affected significantly the number of individuals of small species (repeated measures ANOVA; F_64,7_ = 3.25, p = 0.02), but the abundance varied between dates in an inconsistent manner. Analyses of cumulative data indicated that large-bodied carabids (> 8 mm) were more common in late grazing (F_1,8_ = 15, p = 0.02) and that other species were not affected by grazing regime.

In Pustnäs, beetles were collected only during the second half of the summer. During that period, larvae hibernating species were dominant as in Harpsund, but no significant differences between grazing regimes were found. The number of individuals of small species (< 5 mm) before late onset of grazing significantly differed between grazing regimes at some sampling occasions (repeated measures ANOVA; interaction grazing/time F_26,1_ = 5.5, p = 0.04) and was generally higher in continuous grazing (grazing as main effect, F_26,1_ = 7.1, p = 0.08). After late onset of grazing, the number of individuals of large species (> 8 mm) were significantly more common in late grazing (F_68,1_ = 14.4, p = 0.02). Intermediate-sized species (5–8 mm) were affected by the grazing regime/date interaction (F_68,4_ = 2.9, p = 0.03), and the number of individuals was lower in late grazing at most dates.

**Figure 7.**
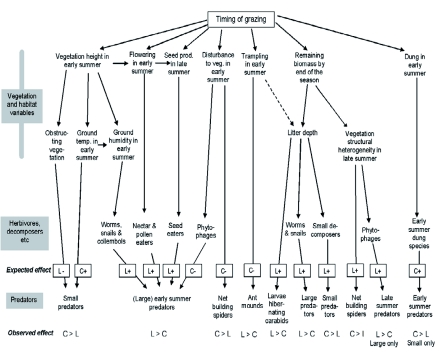
Possible effects of timing of grazing on vegetation and other habitat variables in semi-natural grassland that may affect arthropod predators either directly or through effects on their prey communities. Dashed arrows show weaker effect. The expected effect of two grazing regimes: continuous grazing (C) and late onset of grazing (L) is indicated as either positive (+) or negative (-). The observed effect refers to significant differences in abundance between the two grazing regimes. High quality figures are available online.

Responses of species with different food or habitat preference: almost all species were predators according to literature, and differences between food preference groups were thus not possible to test.

In Harpsund, habitat preference contributed to explaining differences in abundance between grazing regimes. Before onset of late grazing, the number of individuals of species preferring shade or moderate exposure was significantly higher in late grazing sampling of the downhill blocks (F = 8.35, p = 0.04). Also, the number of individuals of species preferring sparse vegetation, differed between grazing regimes at some sampling occasions, but the results varied in a inconsistent manner between sampling dates.

In Pustnäs, the numbers of individuals belonging to different habitat preference groups were low, and no significant differences between grazing regimes were detected.

## Discussion

Open grasslands are exceptionally rich in species from several taxonomic and ecological groups. Most grassland habitats in the temperate regions need grazing, mowing or other types of management in order to persist. Today, much research and conservation work aims at designing grassland management that preserves the grasslands' threatened flora and fauna (e.g. Myers 1998; Kleijn and Sutherland 2003). Timing of management is one aspect that can be easily manipulated for conservation purposes. Several studies have demonstrated effects of timing on growth, flowering, and fruit production of the vegetation-forming plant species (e.g. Wissman 2006). As a consequence, timing affects species groups directly associated with these vegetation features, for example phytophagous insects, nectar- and pollen-eaters, and seed predators (Westrich 1996; Morris 2000).

This study also shows that arthropod predators that are not directly dependent on the vegetation and plant species, are strongly influenced by the timing of management, in this case timing of grazing. In summary, small ants and spiders were in general more common in continuous than in late grazing, whereas larger spiders and *Formica*-ants were more abundant in late grazing. Ant mound density was higher in late grazing in one of the grasslands. The abundance of carabids was higher in continuous grazing in the early summer, but higher in late grazing in the later summer.

The results indicate that timing of grazing affects several habitat variables of the grassland habitat, and that different groups of arthropod predators are reacting to different variables. The possible relationships between timing, habitat variables, and species groups are discussed below and summarized in Figure 7.

### Height and structural heterogeneity of the vegetation

Tall vegetation in late grazing provides a three-dimensional space for climbing arthropods, and this aspect of the vegetation is one conspicuous difference between continuous and late grazing in the early summer. Also in the late summer, patches with tall vegetation were more common in late grazing, as indicated by error bars in Figure 7. Such patches thus formed a higher structural heterogeneity in late compared to continuous grazing (see Pihlgren 2007). Vegetation structure has been shown to be important for web-building spiders (Roberts 1996; Dennis et al. 2001), but contrary to expectations, web builders were more common in continuous grazing. This may be because the grazing cattle destroyed the webs and forced the spiders to move.

In contrast, tall vegetation is an obstructing structure for species running on the ground, in particular predators using visual hunting (Cole et al. 2005). This may partly explain why small running spiders, small carabids, and small ants were more abundant in continuous grazing, although higher microhabitat temperature, as discussed below, may be a more important factor for the small arthropods. Large running predators among all studied groups were more common in late grazing, which indicates that the advantage of larger food supply (see below) is a more important factor for larger arthropods than the disadvantages of obstructing vegetation (Heck and Crowder 1991).

### Temperature and humidity of the microhabitat

Earlier studies have shown that the activity or abundance of small arthropods, in general, can be related to the differences in temperature and humidity caused by different vegetation height (e.g. Treweek et al. 1997). Clapperton et al. (2002) showed that soil temperature was about 5° C higher in a grazed pasture than in a non-grazed pasture, mainly due to shading by tall vegetation. In this study, the average vegetation height in continuous grazing was low throughout the season, while late onset of grazing allowed the vegetation to double in height from June to mid-July. Small poikilothermic animals in cold climates may need to spend more time in warm, sunny microhabitats than larger animals that can spend longer time in colder microhabitats after loading heat in the sun (Sota et al. 2000). In this study, small carabids and spiders were significantly more common in low vegetation (continuous grazing) in the early summer in one of the grasslands, but not later when the vegetation was low in both treatments. The result was thus consistent with expectations, and the preference for low vegetation may have been further enhanced by the temperature changes during the summer. The temperature difference between tall and low vegetation can be assumed to be more important if the general air temperature is low. Temperature data (Ultuna Climate and Bioclimate Station, unpublished data) showed that May and June were considerably cooler than July and August. During May and June, only two out of six ten-day periods had a mean temperature > 15° C, compared to six out of six periods during July and August.

The preference of small carabids for continuous grazing may also be explained by a combination of temperature, body size, and life cycle. The small carabids found in this study were mainly adult hibernating and were thus present as imago in the grassland in the early summer. Larva hibernating species are larger and were emerging as imago from approximately 1 July. Higher abundance of carabids in continuous grazing in the early, but not in the late summer, may thus be because early-summer species are smaller and therefore prefer low vegetation. It is notable that late grazing became more attractive to carabids around 1 July, three weeks before late onset of grazing, but approximately when adult- hibernating species were replaced by larva-hibernating ones.

Temperature and humidity can also be expected to affect the abundance of several of the organisms serving as a prey resource for the studied predator groups. For example, snails and worms are sensitive to desiccation (Andersen 1997) and should be more abundant in late grazing. This may explain the higher abundances of large species of carabids, ants (*Formica* spp.), and spiders in late grazing.

### Food resources as a result of growth, flowering, and seed production

A considerable proportion of the arthropods in a grassland are dependent on the grass sword's plants as their main food resource, and these herbivores comprise a food resource that can be expected to attract predators from all of the studied groups (Morris 2000). The plants are utilized by phytophages, eating plant tissue, sap suckers, pollen eaters, nectar eaters, and seed predators. Most species of these groups of herbivores are more abundant in undisturbed compared to grazed vegetation (e.g. Andrzejewska 1971; Bestelmeyer and Wiens 1996; Treweek et al. 1997; Schwab et al. 2002), especially species feeding on plant reproductive organs or other apical tissue that is frequently removed by grazing (Morris 1967). The relationship between vegetation, prey supply, and abundance of large predaceous arthropods has been found in several studies, for example by Cole et al. (2005), showing fields with tall vegetation having higher abundance of large beetles and wolf spiders, and Dennis et al. (2001), demonstrating higher densities of larger Lycosidae spiders in non-grazed than in grazed fields.

This study confirms these results, as higher abundance of many predators, especially large species, was found in late grazing. In the early summer, the vegetation was undisturbed in late grazing, but also after late onset of grazing, patches of tall vegetation remained for a long time, potentially increasing the abundance of prey herbivores. Among ants, species forming large colonies, such as *Formica* and *Lasius* ants (Hölldobler and Wilson 1990; Lenoir 2002, 2003), can be expected to be highly dependent on large food resources (Petal 1974, 1978). This is consistent with the observation in this study that two *Formica* spp. were more common in late grazing. The abundance of carabids was significantly higher in late grazing after 1 July in both grasslands. As discussed above, the late-summer fauna of carabids consists mainly of large species that may be less dependent on warm microhabitats and more dependent on large food supply, particularly of worms and snails (see Brose 2003).

### Litter

Grazing affects the thickness (Rosén and Bakker 2005) and quality (Bardgett et al. 1998) of the litter layer, mainly through the amount of biomass left after the grazing season, but to some extent by trampling of the litter layer the following early summer (Wissman 2006). This study showed that the slightly taller vegetation in late grazing (about 1 cm of difference) resulted in about 4 mm and significantly thicker litter layer the following spring. Although this difference is small, it may imply significantly larger food resources (Bestelmeyer and Wiens 1996) in terms of Diptera larvae and Collembola (Tian et al. 1993), and snails (Kappes et al. 2005), for example. In Pustnäs, the abundance of earthworms tended to be higher in late grazing (unpublished data). These food resources add to the herbivores discussed above, further increasing the food supply in late grazing.

For carabids, the litter layer may also affect the habitat's suitability for hibernation, which may be part of the explanation for higher abundance of larva-hibernating species in late grazing. Larvae hibernating species hibernate in or close to the foraging areas (Lindroth 1992). Although not studied, a thicker litter layer and a more heterogeneous vegetation structure may provide favorable conditions for hibernation (cf. Brose 2003, MacLeod et al. 2004). If so, adult carabids can be expected to be more common in late grazing, both because they are hatched there and because they may choose habitats that are optimal for larvae hibernation. In contrast, adult hibernating species migrate to suitable hibernation sites, sometimes far from the summer foraging areas (Lindroth 1992). In the spring they migrate back, possibly choosing the optimal foraging and breeding habitats.

### Competition

Some results of the current study may be explained by competition. For example, *Formica* ants are known to compete with other ant species for food and suitable nest sites and also to affect other species by predation. Lesica and Kanowski (1998) showed that *Formica* and *Myrmica* ants compete for suitable nest sites, resulting in a lower nest density of *Myrmica.* Activity of *Leptothorax* and *Myrmica* ants has been shown to be higher when *Formica* spp. were absent (Puntilla et al. 1991). In the current study, the presence of *F. polyctena* and *F. rufibarbis* in late grazing may have suppressed the activity of *Myrmica* spp.

The colonies of *Formica* ants may also have had a negative effect on the Linyphiidae spiders. Lenoir (2003) found that wood ants that were manipulated to forage exclusively on the forest floor had a negative effect on the activity of Linyphiidae. However, web-building spiders can escape from interference with ground-dwelling wood ants by ‘staying by their webs’ (Lenoir 2003). In the present study, activity of arthropods was measured by the use of pitfall traps, which may reflect higher rates of web destruction. Since no data on abundance of spider webs was collected, it was not possible to estimate the actual abundance of Linyphiidae.

### Dung deposition

Dung serves as an essential substrate for a number of obligate coprophilous arthropods, of which some can be expected to serve as prey for the studied predator groups. Some groups of small arthropods followed temporally the distribution of dung, i.e. were more common in continuous grazing in the early summer and equally common in the two treatments after late onset of grazing. It is possible that dung is important for small predators, but its effect can not be separated from the effect of low vegetation and sun exposure, as discussed above. No groups of larger predators followed the abundance of dung, and it is likely that the effect of dung on the food supply for large predators was of little importance compared to the effect of vegetation height and growth as discussed above.

### Mechanical disturbance by the grazers

The grazers cause mechanical damage and disturbance to the grassland ecosystem, mainly by grazing and trampling. This presumably affects the studied groups of arthropods both directly and indirectly. The main, indirect effect of trampling and grazing (Zahn et al. 2007) is reduction of prey populations, for example by trampling of ground fauna and grazing of sessile life stages of phytophages and seed predators (Zahn et al. 2007).

Direct effects of disturbance are trampling mortality of ground dwelling specimens, damage of spider nets, and damage of ant mounds. For example, Duffey (1975) showed that abundance and diversity of spiders was reduced by trampling by cattle. In this study, larger running spiders were less common in continuous grazing, but, as discussed above, this may be primarily an effect of larger food supply in late grazing. It has been shown that some mound-building ant species are sensitive to grazing, probably due to trampling by the grazers (Beever and Herrick 2006). In the current study *L. flavus* was present in large numbers in late grazing in Harpsund, while it was almost absent in continuous grazing. In Pustnäs, cattle were observed destroying ant mounds of *L. niger*, but there were no such observations from Harpsund.

### Synergies and tradeoffs between habitat variables

Some results of this study indicated that two or more of the discussed habitat variables may have synergistic effects on predator abundance, whereas other variables may have opposing effects. Of the variables discussed here, some can be assumed to affect habitat choice of predator arthropods by creating attractive conditions in the grazing treatment in question; other variables have a repelling effect, thus decreasing the abundance in the treatment (Figure 7). One example of a possible synergy is that both favourable temperature conditions (in continuous grazing) and unfavourable hunting conditions (in late grazing) can be expected to increase abundance of small predators in continuous grazing. Another example is that both litter and tall vegetation in late grazing may increase prey abundance, thus attracting predators to the treatment area. For carabids, the effect of litter on hibernation conditions may act in synergy with the two mentioned variables.

One example of a possible trade-off between variables is that higher prey abundance attracts while colder microclimate repels small predators in late grazing. In this case, the effect of microclimate seemed to be more important. Another example of opposing effects is that destruction of ant mounds may repel and higher temperature may attract small ants (*L. flavus*) in continuous grazing, while larger food resources may attract the ants in late grazing. In this case, destruction of mounds and food resources seemed to be more important than a warmer microclimate.

### Methodical comments

Ideally experiments with grazing regimes should be replicated across a number of sites. Marriott et al. (2004) reviewed many site-specific effects and suggested that replication would allow the extraction of general principles from the data. However, replication of grazing treatments are very costly if the treatment areas and cattle groups are as large as in this experiment, allowing the cattle to express a natural grazing behaviour. This setup thus mimics practically applicable grazing regimes, but requires the use of two large treatment areas per site instead of a number of small, interspersed areas. Small treatment areas would create artificial conditions because when small late-grazed exclosures within continuous grazing areas are opened for the grazers, the vegetation is grazed much faster than in a large late-grazed area (unpublished data). Also edge-effects can be expected when grazing exclosures are small and fast running carabids and wolf spiders might either just run through these exclosures or accumulate for shelter. Trap records will, therefore, not provide data on community density of these animals.

An obvious disadvantage with the chosen experimental design is that it raises a pseudoreplication problem in several types of statistical tests (Hurlbert 1984; Oksanen 2001; Hurlbert 2004; Oksanen 2004). The data collected at one site were, in fact, two random samples from two different areas, being treated in two different ways. Due to this, and due to the differences in data sampling design between the two grasslands, all analyses were performed for each grassland separately. Furthermore, the results must be interpreted acknowledging the possibility that the observed differences were area effects rather than treatment effects.
